# AI under great uncertainty: implications and decision strategies for public policy

**DOI:** 10.1007/s00146-021-01263-4

**Published:** 2021-09-07

**Authors:** Maria Nordström

**Affiliations:** Division of Philosophy, KTH Royal Institute of Technology, Stockholm, Sweden

**Keywords:** Artificial intelligence, Public policy, AI policy, Uncertainty, Great uncertainty

## Abstract

Decisions where there is not enough information for a well-informed decision due to unidentified consequences, options, or undetermined demarcation of the decision problem are called decisions under great uncertainty. This paper argues that public policy decisions on *how* and *if* to implement decision-making processes based on machine learning and AI for public use are such decisions. Decisions on public policy on AI are uncertain due to three features specific to the current landscape of AI, namely (i) the vagueness of the definition of AI, (ii) uncertain outcomes of AI implementations and (iii) pacing problems. Given that many potential applications of AI in the public sector concern functions central to the public sphere, decisions on the implementation of such applications are particularly sensitive. Therefore, it is suggested that public policy-makers and decision-makers in the public sector can adopt strategies from the argumentative approach in decision theory to mitigate the established great uncertainty. In particular, the notions of framing and temporal strategies are considered.

## Introduction

This paper will discuss decision-making on public policy for artificial intelligence systems (henceforth “AI”) in the public sector. I argue that decisions regarding AI policy are decisions under *great *uncertainty. Uncertainty warrants consideration in the context of public policy due to the obstacles it can pose to effective decision-making if ignored or misunderstood (Nair 2020). Moreover, not adopting relevant policy strategies in a timely manner and subsequent delays can lead to high societal costs (ibid). Decision-makers underestimating the magnitude and range of uncertainties can lead to policy failure. Given that the uncertainty regarding policy on AI is classified as ‘great’, it cannot be expected to get resolved with time or new information. Therefore, I discuss strategies to account for and mitigate the uncertainty. Namely, I suggest that decision-makers can incorporate elements from the argumentative approach in decision theory (Hansson and Hirsch Hadorn 2016)^1^, a pluralistic analysis of the normative issues involved in decision-making to better understand the uncertainties involved in a decision. In particular, decision-makers may find temporal strategies useful to ensure an adequate mode of procedure. Additionally, I argue that decision-makers need to be aware of the role of framing when it comes to policy on AI for public use^2^, specifically in light of great uncertainty.

While the uncertainty as such does not stem from any specific sector or application of AI, I argue that there is additional responsibility for implementations of AI for public use to ensure that principles of democracy are being upheld. Some of the most problematic uses of automation concern functions central to the public sphere and need careful consideration (Smuha 2020). Hansson and Hirsch Hadorn have suggested that the argumentative approach is beneficial for democratic decision-making where the decision support should enable decision-making with democratic legitimacy (ibid). Hence, the argumentative approach and its components are highly relevant in the context of decision-making regarding AI.

The scope of decisions an AI can make seems endless with systems determining choices, opportunities and legal positions of certain sections of the public (Harkens 2020). Given the significance of such decisions, concerns have been raised regarding privacy, safety and security, transparency, bias and fairness, among other issues (Coeckelbergh 2020). Furthermore, agreeing on a definition of AI is difficult, indicating that we do not fully know what to expect of AI. It has been said that AI systems “present exceptionally broad and intractable uncertainties about their benefits, risks, and future trajectories” (Wallach and Marchant 2019, p. 505). Simply put, “impacts [AI] can have on us as individuals, groups and societies – and particularly the negative ones—in the shorter and longer term are still uncertain and not yet fully understood” (Smuha 2021). In the recent Policy and Investment Recommendations for Trustworthy AI by the EU Commission’s High-Level Expert Group on Artificial Intelligence, it is acknowledged that there is little evidence to inform policy-making “due to the novelty of the technology, the lack of thorough and systematic understanding of its impacts and associated business models, and the unpredictability of its uptake, development and evolution even in the short term” (2019). Considering this, I argue that decisions regarding AI cannot rely on traditional, reductive decision theory with a clear set of options and outcomes. Decision-makers who fail to correctly acknowledge uncertainties and their implications as well as the limits of available information might fail to be flexible and adapt when needed.

### Preliminaries

There does not seem to be a generally accepted definition of AI [the difficulty of defining AI is developed in Sect. 4 (i)]. For the purposes of this paper, AI is to be understood as data processes that interpret particular input and carry out operations (sometimes according to specific instructions) that would require cognitive functions when done by humans or intelligent beings, with the aim to achieve a particular goal.^3^ Further, AI is conceptualized as programs and systems working towards achieving an optimal result, and in case of uncertainty, the best expected outcome within a predefined set of boundaries and set of rules by learning from previous experiences. Even though the intent is to discuss AI systems, i.e., tangible technology, compared to AI, which is often understood to be more of a general concept, I will henceforth use ‘AI’ and ‘AI system’ interchangeably in reference to ‘AI systems’, for the sake of readability.

Lastly, I need to establish further delimitations. This paper focuses on *decisions regarding public policies for AI* and not decisions made by an AI as such. Moreover, the intent is to consider narrow AI,^4^ even if some of the reasoning could be applied to general AI.

### Outline

The rest of this paper is structured as follows. In Sect. 2, I present the notion of uncertainty and decisions under great uncertainty. Section 3 briefly covers uncertainty regarding technological advancements. I argue that decisions on policy regarding AI are decisions under great uncertainty in Sect. 4 and discuss public policy and AI in the public sector in Sect. 5. The implications of the established uncertainty are considered in Sect. 6. Therefore, I suggest two elements of the argumentative approach that could be useful to mitigate the great uncertainty of policy on AI in Sect. 7. Finally, I conclude in Sect. 8.

## Decisions under great uncertainty

There is a vast body of literature on the concept of uncertainty^5^ in disciplines such as social science, economics, philosophy, and psychology, which may influence policy theory and science (Nair 2020). For example, the notion of uncertainty has been said to pose a fundamental challenge in economic theory as it both complicates the decision-making process and calls into question the “optimizing assumption itself” (Beckert 1996). In decision theory, a common assumption is that decisions are based on values or criteria that are “well-defined and sufficiently precise” (Hansson and Hirsch Hadorn 2016). In practice, this is often impossible. Decisions are then taken under varying degrees of uncertainty. The distinction between risk and uncertainty was originally made by Knight (1921); decisions under risk being decisions where the decision-maker knows the probability of the possible outcomes, whereas the decision is said to be under uncertainty (sometimes called ‘under ignorance’) if the probabilities are unknown or nonexistent. There is also plenty of situations where the uncertainty goes beyond probabilistic uncertainty, for example, when potential outcomes, the alternatives the decision-maker can choose between, what the problem to be decided on consists of or what aspects to include, are not known. Decision problems where multiple such circumstances prevail can be called decisions under ‘great’ uncertainty. Related terms are ‘fundamental’, ‘radical’ or ‘deep’ uncertainty, all of which refer to complex situations where information for a well-informed decision is lacking to some degree.

Another type of similar problem is called ‘wicked’. Rittel and Webber originally identified ten primary characteristics of wicked problems (1973), problems that are complex, unpredictable, open-ended, or intractable. Though the initial definition by Rittel and Webber is somewhat vague, more recent attempts have been made to decompose wicked problems into more nuanced categories to understand better what makes such problems challenging for policy-makers (Head and Alford 2015). Head and Alford argue that there are degrees of “wickedness” and that while conclusive solutions are rare, finding partial, provisional courses of action is possible. However, the term ‘wicked’ can evoke certain associations of intent and ‘evilness’, associations that terms such as ‘great’ and ‘deep’ avoid. Still, problems denoted as wicked share policy-relevant features and pose similar challenges as decision problems under ‘great’ or ‘deep’ uncertainty. For example, wicked policy problems are said to be difficult to identify and interpret: “the evidentiary and the interpretative elements of policy analysis become increasingly indistinguishable and inseparably intertwined” (Daviter 2019). Such policy problems remain fundamentally ambiguous and resist standard approaches to problem-solving, according to Daviter, who argues that the role of knowledge in this context has to refer to the interpretative basis of policy inquiry along with the evidentiary basis of analysis.

Efforts to refine the concept of uncertainty have been made in multiple disciplines, such as in international relations, decision theory, and economic theory. For instance, there can be a distinction between ‘ontological’ uncertainty and ‘epistemic’ uncertainty. The term ‘epistemic uncertainty’ has been used to refer to both uncertainties due to limitations in people’s abilities and uncertainties due to the unpredictability of future events (Dequech 2004). ‘Ontological uncertainty’, on the other hand, is used to describe some properties of reality. Dequech argues that the concept of uncertainty has both ontological and epistemic dimensions. It is always associated with a lack of knowledge and with an associated view of reality. In international relations, different meanings un uncertainty can be elicited depending on the paradigms of realism, rationalism, cognitivism and constructivism (Rathbun 2007). In the work of Rathbun, the respective conceptions of uncertainty are understood to be fear, ignorance, confusion, and indeterminacy. For example, in the paradigm of cognitivism, uncertainty is conceptualized as confusion, a function of the unclear signals that policy-makers are receiving.

In political analysis, Nair recently summarizes frameworks for categorizing uncertainties, for example based on the object of uncertainty and approaches to address the uncertainties (2020). For example, ‘epistemic’ uncertainty is a lack of knowledge about the substance of the issue when the object of uncertainty is substantive whereas ‘ambiguity’ is due to different frames about the substance of the issue. However, many frameworks do not distinguish between types of uncertainties that are irreparable and those that can be ‘solved’. A framework that considers this distinction is a recent approach to consider the quantifiability and formalizability of uncertainty by Hansson (in press). He develops a refined topology of types of uncertainty, including:

- ‘Factual uncertainty’ as uncertainty about states of the world,
- ‘Possibilistic uncertainty’ as uncertainty about what has been, is or will be possible,
- ‘Interactional uncertainty’: uncertainty about interactions with other agents,
- ‘Value uncertainty’: uncertainty about one’s values,
- ‘Structural uncertainty’: uncertainty about the structure and delimitations of the decision problem,
- ‘Linguistic uncertainty’: uncertainty about the meaning of linguistic expressions.

The topology provides a structure that is helpful to clarify if different uncertainties can be removed, whether it will require time or resources, or if certain uncertainties cannot be reduced. For example, ‘factual uncertainty’ might be resolved with more information. Yet, knowledge or facts about the world will not necessarily reduce other types of uncertainty, such as ‘possibilistic uncertainty’ or ‘structural uncertainty’. Similarly, while efforts to agree on precise definitions can reduce ‘linguistic uncertainty’ and ‘structural uncertainty’ to some degree, such efforts do not commonly reduce ‘value uncertainty’. The analysis of this paper will apply the topology by Hansson in order to characterize the uncertainties in decisions on AI policy.

## Uncertainty and technology

It could be argued that all technological developments are highly unpredictable and therefore, policy-makers face the same difficulties when establishing regulations and policies concerning all technology. For example, technological innovation (together with managerial or organizational innovation) is by Dequech argued to be the best example of unpredictable structural change: “if technological innovation is properly considered, then the uncertainty associated with it is of the fundamental kind” (Dequech 2004). While the development of science and technology is sometimes unpredictable, a general unpredictability claim is exaggerated. Policy measures and investments into specific technology tend to generate the expected technological advancements. Some technological advancements are based on incremental, gradual improvements of current technology, for instance, the combustion engine and computer hardware performance. Carrier argues that “[s]uch technological changes proceed in smooth transitions whose likely future can be foretold without much uncertainty” (Carrier 2019). A particularly relevant aspect is that while the technological development can be predictable, the societal factors and affecting the technology (and affected by the technology) are much more difficult to predict (Hansson 2011). The telephone and the Internet are examples of such technological advancements where the impact was not foreseen. Can we a priori know if the impact of a new technological advancement will be difficult to foresee, like the Internet, or expected and incremental, like the combustion engine? For the sake of the argument in this paper, we do not have to. Not every technology that requires policy concern is equally (greatly) uncertain in aspects that are relevant for policy-makers. Some scenarios are more probable than others, “[t]his suggests that we are able to foresee the future course of science and technology in a coarse-grained and defeasible manner” (Carrier 2019). The development of batteries is likely to yield less policy-relevant uncertainty than agricultural biotechnology. For reasons developed below, AI has a significant level of uncertainty which is relevant to policy, similarly to for example policy on climate change or biodiversity (Polasky et al. 2011; Haila and Henle 2014).

## Are decisions on AI policy decisions under great uncertainty?

Below, I will consider the various types of uncertainty prevalent in the context of policy regarding AI. I base these considerations on three features of the policy problem at hand: (i) vagueness of the definition of AI, (ii) uncertain outcomes of AI implementations and, (iii) pacing problems. These features give rise to possibilistic, structural, linguistic, and interactive uncertainty, which I argue are sufficient to establish that great uncertainty prevails.

(i) Vagueness of the definition of AI

As mentioned in the introduction, the difficulty of precisely defining what AI is has been brought up as an obstacle to effective regulation (Scherer 2015). Any regulatory regime must define what it regulates precisely, argues Matthew Scherer. Since there is not a widely accepted definition of AI, any regulation of AI must for now be limited. John Danaher argues that the vague definition of AI is not necessarily an obstacle since there are other vague concepts that we have managed to regulate, such as ‘energy’ and ‘medicine’ (Danaher 2015). While policy itself cannot be vague, it can clarify vague concepts and ensure that the ‘vagueness’ is captured in the regulatory process. Still, a relevant concern is that a focus on solutions made possible by AI skips the stage of problem structuring and definition (Veale 2020). This is particularly true when AI is deployed to solve a problem with no agreement on the means and end goals. The ‘magic’ of AI (as discussed by Elish and Boyd (2017)) might make it seem like such an agreement is not necessary. However, without knowing the proper scope, a decision-maker cannot know the demarcation of the problem nor put together a list of options to consider. Thus, the vagueness of the definition gives rise to so-called *structural uncertainty*, i.e., uncertainty about the structure and delimitation of the decision. Structural components are here assumed to be the scope of the problem, the appropriate subdivision, the body responsible for the decision, the options, timing, the appropriate horizon, and framing. Moreover, the vagueness of the definition also gives rise to a *linguistic uncertainty*, i.e., uncertainty about what is *actually* meant by the expression used. This type of uncertainty can stem from both ambiguity and vagueness. Lastly, the definition of AI clearly influences what can be counted as ethical issues arising from AI.

(ii) Uncertain outcomes of AI implementations

Not only do we not know how general AI will impact society, but we also cannot be sure of the consequences of a narrow AI implementation. This is due to the very nature of AI: “AI systems are often designed to be autonomous and to act in creative ways (i.e., ways that are not always reasonably foreseeable by the original designers and engineers)” (Danaher 2015). Hence, there is inherent *possibilistic uncertainty*, uncertainty about what is and will be *possible*. While this is a type of epistemic uncertainty, it is substantially different from *factual uncertainty* since it is one thing not to know what is and another not to know what is possible. This type of uncertainty is related to the difficulty to foreseeing new technological effect: effects of new technology are often determined by chains of events that no one had thought of beforehand (Rosenberg 1995). For example, an algorithm can identify a pattern that the original decision-maker did not intend to guide its decisions. As pointed out by Renda “[t]his does not imply that AI is developing its own intelligence that departs from the goals and tools given to it by developers: however, these techniques instill an element of randomness and uncertainty in the way machines use data to reach optimizing decisions” (Renda 2019). It can be argued that this uncertainty should be easy to counter ex post, i.e., after a decision is taken, the algorithm has to include measures to explain its decisions in order to ensure transparency. This is often referred to as *explainable AI* (XAI) and why the virtue of transparency is vital in many AI policies brought forward (Lepri et al. 2018).^6^ It might be possible to reduce the ex post uncertainty to a certain degree by explainable AI (Biran and Cotton 2017). However, the sufficiency is so far disputed.

Furthermore, the uncertainty regarding the grounds on which a decision is made persists if the decision is not explainable. Even if the mechanism behind the decision-making is explainable there is still another issue, namely the ‘foreseeability problem’. Regardless of a potential ex post explanation, we cannot ex ante predict the consequences of an AI application: “we cannot know for sure that a given [AI] application is safe unless we can test the application in all possible contexts” (Floridi et al. 2020, p. 5). Considering this impossible, Floridi et al. argue that complete certainty is out of reach and that what we in fact have is “an uncertain and fuzzy world with many unforeseen situations” (ibid). While the difficulty to forecast outcomes is not unique for AI, it is arguably significant for AI. Not only are the consequences not known, it is also difficult to determine how probable different possible outcomes are or what outcomes are possible at all.

(iii) Pacing problems

There is a relatively low threshold to deploy an AI solution with promises of great reward in terms of efficiency, leading to one major concern, namely the so-called ‘pacing problem’. Multiple authors have argued that policy-makers struggle to ‘keep up’ (Cath 2018; Wirtz et al. 2019; Reinecke et al. 2021). The concern is that the technological advances are so fast that regulations risk being obsolete once implemented. This reasoning suggests that traditional means of regulation are inadequate, opting for ‘soft law’ regulations instead (Wallach and Marchant 2019). Soft law is substantive expectations that are not directly enforceable, such as principles, codes of conduct, best practices, and guidelines. The proponents of soft law believe it has the benefit of being possible to adopt and revise more quickly (ibid). It also has the benefit of addressing technology holistically and involving a broad range of stakeholders using a so-called cooperative approach. However, there is a lack of enforceability, so, in time, soft law would have to be implemented as traditional regulations. Another disadvantage is the multitude of initiatives to develop suitable soft law, i.e., principles and codes of conduct. Just the effort on keeping up with the development of principles and guidelines can be a challenge (Jobin et al. 2019).^7^ In their paper, Jobin et al. identified 84 various AI ethical principles of guidelines released by (among others) academia, governmental agencies, private companies, and NGOs (ibid). As Cath writes: “[a]cademics and regulators alike are scrambling to keep up with the number of articles, principles, regulatory measures and technical standards produced on AI governance” (Cath 2018). Decision-makers do not only have to manage uncertainties with regards to the technology itself and the rapid speed of development; they can also be expected to keep up with the rapidly developing policy sector. Additionally, AI will most likely be developed by the private sector, assumably at high speed. Hence, policy-makers can be said to be dependent on the decisions of others, a type of *interactive uncertainty*, uncertainty due to unknown choices and actions by others. According to Hansson (in press), this type of uncertainty is common in professional and large-scale social activities where there might be uncertainties concerning social interactions.

Does this equal great uncertainty?

The analysis above establishes that there is possibilistic, structural, linguistic, and interactional uncertainty related to AI policy. Even if, by certain measures (such as definitions, and collaboration agreements), the linguistic, structural, and interactional was resolved (though, given the complexity of the problem, fully resolving the structural uncertainty seems unlikely), the possibilistic uncertainty will remain. Hence, the uncertainty is arguably ‘great’. While Andrews argues that so-called algorithmic issues are not ‘wicked problems’ since regulatory bodies are taking on the issues and there are apparent solutions, the argument concerns specific challenges such as selection error, law-breaking, manipulation, and propaganda (Andrews 2019). These concrete issues do not capture the broad uncertainty of the societal implications of AI (Andrews notes that algorithms that challenge human comprehension could indeed be ‘wicked problems’).

Moreover, while it can appear to be less uncertainty for narrow AI than general AI, we arguably do not know the potential of AI and thus our actual options (at least from a policy perspective). While we can apply technological assessment and forecasting techniques, we are still fumbling in the dark. Assuming there is only ‘factual uncertainty’ can lead to unnecessary delays ‘until we know more’. Such delays can have grave societal costs and not lead to better decisions since other types of uncertainties will not be resolved by additional knowledge. Given the great uncertainty, the cost of acquiring more information to achieve an improved forecast might by far exceed the profit to be expected in the form of a better decision (Gärdenfors 1979).

## AI and public policy

The rapid technological development and societal implications of AI pose a challenge for policy-makers. The need for regulation of AI systems has been widely recognized; it has even been argued that the ‘race to AI’ brings forth a ‘race to AI regulation’ (Smuha 2021). This is not to say that all AI systems and algorithms ought to be regulated under the same principles. Smuha notes that the term ‘regulating AI’ can make it seem like the same regulatory measures are equally applicable and relevant in all situations, but this is not the case; context matters. The regulation of AI must be sector-specific (Nitzberg and Zysman 2021). For example, there might be one approach to AI regulation in the financial sector (Truby et al. 2020) and another approach to AI in healthcare (Sharma and Manchikanti 2020). However, some policy features might span over multiple sectors, such as for regulation to be proactive and responsive. This is due to many of the regulatory challenges being similar. Matters of risk and liability (of AI-caused harm) and concerns regarding AI not respecting values such as autonomy, fairness, and privacy, have been considered (Scherer 2015, among others). Yet, Perry and Uuk claim that the amount of work that has been done on developing policy solutions to AI risk is modest, with most of the efforts in the context of general AI (Perry and Uuk 2019).^8^ If the efforts are indeed modest, then the main interest being on policy for general AI can be understandable. After all, an actual ‘superintelligent’ AI will require that regulating and policy-making bodies ensure “that AI is developed, deployed, and governed in a responsible and generally beneficial way” with potential risks in mind (Bostrom et al. 2020). However, the governance of near-term (or even currently deployed) AI for public use is undoubtedly more pressing and is getting recognition (Andrews 2019). Wirtz et al. provide a review of the current state of research on AI in the public sector, including various types of applications and the respective challenges but note that there is little knowledge on the types of possible AI applications and the overall potential of AI for governments (Wirtz et al. 2019).

Why is there a need for specific policy for AI at all? There seems to be an underlying mantra: “AI is different: it is not like the Internet, not like electricity, not like the industrial revolution, not like oil and not like the invention of the wheel” (Renda 2019). Further, “so the gospel goes, we need new laws, new rules of conduct, new criteria for interacting with machines and a lifeline in case they decide to take over” (ibid). Is there a policy vacuum or a lack of appropriate regulation due to fast technology development? Bostrom et al. do argue that in the context of general AI, the development will be transformative enough to set unique policy challenges and note that “[t]he context of a machine intelligence revolution would place unusual epistemic demands on the policymaking process” (ibid). They claim that the challenges that the decision-makers face in this context involve deep, fundamental empirical and philosophical questions clouded in uncertainty. Additionally, they note that it is a matter of pace with governmental processes having to be more rapid than usual and operate on much shorter timescales. However, the need for speedy governance also occurs in other areas of policy-making. The challenges of the covid-19-pandemic have forced policy to be established very rapidly and require constant adjustments, also under uncertainty (Ongaro 2021). Any type of rapid development with substantial societal consequences requires a particular type of approach to public policy. In this context, it is unclear why policy on general AI would differ from policy on other quick and fundamental developments. For example, Joanna Bryson argues that AI as a technology is not as unusual as expected with quite familiar challenges at hand (Bryson 2019). She adds that it might still require radical innovations in the ways we govern.

Similarly, Elish and Boyd point out that in many regards, there is nothing new about either Big Data or AI (Elish and Boyd 2017). Instead, they problematize the myths of the supposed “magic” of these such systems. The hype and promise of AI has led to a rhetoric around the technology that the actual techniques do not live up to. AI already exists in a regulatory framework (Brundage and Bryson 2016). While the promise and challenges of AI are more boring and less disruptive, there is still a great promise (Renda 2019). If this promise is to be realized, the public sector needs to act as a possible driver of innovation. The public sector also needs to function as a platform where the challenge of ensuring a balance between public safety and the essence of our democratic freedoms is recognized. Algorithms are vulnerable to biases in the original data and to making decisions on arbitrary grounds. Unexpected implications of ‘pattern recognition’ can be complicated to safeguard against.^9^ Therefore, public policies are needed to ensure that implementations of machine learning and AI are in line with democratic principles.

### The role of the public sector

As outlined by AI policy documents, the prescribed role of the state is to be active and collaborative in AI development and use (Ulnicane et al. 2020). Additionally, the role of the public sector is explicitly is highlighted in the Policy and Investment Recommendation by the European Commission Independent High-Level Expert Group on Artificial Intelligence (2019). The development and implementation of AI solutions and enablement of digitized public services will assumingly make governments more efficient and help ensure better evidence-based policy decisions. It is suggested in the recommendations that “[h]arnessing the public sector “as a platform” could lead to new opportunities for researchers and entrepreneurs to gain access to data and infrastructure for developing welfare-enhancing AI solutions” (ibid). The public sector thus plays an important role ensuring the adoption of so-called Trustworthy AI (European Commission Independent High-Level Expert Group on Artificial Intelligence 2019) without lowering the quality of human relationships within public services or reducing such services. The recommendations do note that the governments have to safeguard fundamental human rights and protect individuals’ integrity. They should also ensure that individuals are protected from potentially harmful uses of AI. All in all, the recommendation sets quite a challenge when it urges to ensure that AI-based public services are that public services are”deployed for all, and in a manner that safeguards individuals’ fundamental rights, democracy and the rule of law” as well as to within public procurement processes “allocate substantive funding to innovation-driven, AI-based solutions, ensure that potential risks of the use of AI by the government are identified, assessed and appropriately addressed” (European Commission Independent High-Level Expert Group on Artificial Intelligence 2019).

It is acknowledged that establishing appropriate governance and regulatory framework is no easy task, especially since little evidence is available to inform policy-making. Among the guidance on what to consider when formulating new regulations, a principle-based approach (compared to prescriptive regulation) and a precautionary principle-based approach are suggested (ibid). Other scholars have also noted that “the challenges of AI play particularly in the public sector a special role, as the protection […] of citizens and their provision with goods and services they cannot provide on their own is a central part of governmental duties” (Wirtz et al. 2019). Furthermore, given the specific challenges in governments’ use of AI, good oversight procedures are believed to be crucial to ensure that the use of AI is in accordance with collective objectives (Henman 2020).

## Implications of uncertainty for public policy

I have argued that decisions on policy for AI for public use are decisions under great uncertainty. Consequently, more information will not reduce the existing uncertainty. Hence, the uncertainty has to be managed and taken into account during the policy process. Uncertainty as such is common in the context of policy-making; there are problems with systemic complexity, multiple frames, contested policy definitions and contested knowledge, among other related issues (Daviter 2019). However, Sreeja and Howlett state that “the inability to clearly see the horizon of the future policy environment in which impacts of the policy will develop, requires corrective lenses to help clarify and offset the uncertainties with which policy-makers are dealing” in order to not end up with ‘policy-myopia’ and subsequently policy failure (Sreeja and Howlett 2017). Different stages of the policy process are associated with different types of policy failures. For example, at the stage of policy formulation, policy-makers might attempt to deal with ‘wicked problems’ “without appropriately investigating or researching problem causes or the probable effects of policy alternative” (Howlett et al. 2015). The underlying uncertainty needs to be acknowledged, recognizing the challenges it brings forth for policy-makers in general and for AI for public use in particular. Still, uncertainty is not normatively ‘bad’, nor does it imply research or implementations of AI should be stopped altogether. Instead, there needs to be a balance between the potential benefits and risks, including unintended ones. Furthermore, there need to be strategies built into the governance mechanisms that allow for iterative assessment and review to consider and evaluate technological advancements.

## Strategies to mitigate uncertainty

Given that decisions on policy regarding AI are decisions under great uncertainty, it is beneficial to consider how to ensure that proper deliberate procedures taking this into account can be implemented when developing and adopting policy. There is an “ever-expanding suite of approaches, tools and methods” (Nair 2020) for policy-makers to choose from to enable policies to adapt to anticipated and unanticipated changes in the future. Possible strategies include adaptive policy-making, adaptation tipping points and dynamic adaptive policy pathways, among others (ibid). Arguably, the appropriate response depends on the type of uncertainty. There are suggested tools specifically for deep uncertainty suggested by Walker et al. such as multi-stakeholder deliberation, formal policy review and continuous learning (2010). Additionally, given that the uncertainty in policy on AI can be characterized as great uncertainty, it can be worth considering relevant strategies from the argumentative approach (Hansson and Hirsch Hadorn 2016): a pluralistic analysis of the normative issues involved in decision-making. Two such strategies are discussed in detail below.

### Framing

The concept of framing, or frame analysis, is well-established in public policy and is highly relevant in the context of uncertainty: “uncertainty often arises not only due to imperfect information but also due to multiple perspectives and interpretations” (Jones and Baumgartner 2005). However, the term ‘framing’ or ‘framing effects’ is considered in a wide range of academic disciplines and can refer to different phenomena. One understanding of framing (analysis) is as an analytic tool “for those seeking to understand, for instance, issues in the mismatch between administrators’ implementation of legislated policies and policy intent” (Van Hulst and Yanow 2016). This conceptualization of framing or use of framing analysis was originated by Goffman (1974), in whose work ‘framing’ was the answer that explicated the question ‘what is going on here?’. According to Van Hulst and Yanow (2016), Schön and Rein (1994) advocated for “frame reflection” in the policy process, i.e., for policy-makers to reflect on their frames and how they might be contributing to contentious situations. To make the policy analytic focus on framing clearer, Van Hulst and Yanow advocate a shift from ‘frames’ as objects people internally possess and develop for explicitly strategic purposes to ‘framing’—the multidimensional and socio-political processes through which the frames are constructed (ibid). In this sense, framing both organizes prior knowledge and held values as well as guides emergent action.

Additionally, through framing processes, ideas can form public discourse and impact policy development by constructing reform imperatives (Béland 2009). By defining the cause of a problem the solution for the set problem, ideas can enable agents to challenge established institutional arrangements and hence be powerful ideological weapons (Blyth 2001). In framing analysis, a distinction can be made between ‘action frames’ that inform everyday life and policy practice and ‘rhetorical frames’, which refer to the use of story-telling and argument in policy debate (Béland 2009). If there is high uncertainty, existing institutional arrangements are less likely to determine the behavior of key political actors (Blyth 2002). In such situations, two actors can have contrasted views of their interests, even if they occupy the same economic and institutional position (Béland 2009).

In decision theory, ‘framing’ is understood in a ‘strict’ sense, i.e., how the conception of a specific decision problem affects decision-making. In this context, framing refers to a decision problem being formulated in different but logically equivalent ways, and framing is seen as inextricably linked to normative judgment (Grüne-Yanoff 2016). Moreover, one particular way of framing a decision is not necessarily the correct one. For example, consider the description of a glass of water as half-full compared to half-empty. Both these descriptions are ‘correct’ and logically equivalent, yet the descriptions induce different intuitions regarding the status of the water and glass. Here, framing is an important set of phenomena that challenges the standard theories of rational decision-making and the notions of rationality they presuppose. Given a specific account of rationality,^10^ experimentally identified framing phenomena show that people behave irrationally in a systematic way, i.e., changing their preferences based on framing of the decision problem (Tversky and Kahneman 1981). This creates uncertainty regarding individual preferences. Suppose an irrelevant change of frame (i.e., the decision problem is changed semantically but remains logically equivalent) prompts a change in preference. In that case, it creates uncertainty regarding whether the preference is ‘genuine’. Similarly, there is uncertainty regarding the rationality of such decisions since they might be unduly influenced by framing (Grune Yanoff 2016). Given this, it would seem that people need help from the policy-maker to correct their irrational behavior. In turn, the policy-maker can use knowledge on how framing effects influence behaviors and use framing when formulating policy to achieve the desired ends. Framing functions in a justificatory role: “[s]o long as people are not choosing perfectly, it is at least possible that some policy could make them better off by improving their decisions (Sunstein and Thaler 2003). However, the account of it being irrational to decide differently depending on how a decision is framed is not uncontroversial “because the different descriptions of the same fact might convey different information about the expectations of the chooser” (Grüne-Yanoff 2016). Some invariance violations are arguably compatible with a normatively valid model of so-called bounded rationality (Simon 1979). Furthermore, framing understood in the ‘wide’ sense as Goffman and subsequent scholars see framing (analysis) is as sense-making to reduce uncertainty. The presence of frames, attitudes, and contrasting perspectives in the ‘wide’ sense does not serve as justificatory for policy interventions. Rather, it brings forth the need to better under the actors involved. On the other hand, framing in the ‘strict’ sense can still prove to be an effective policy means for the purpose of influencing people’s choices: “[i]n this case, (re-)framing as policy intervention is motivated by the goal to get people to choose what they really want” (Grüne-Yanoff 2016).^11^

In the literature, the concept of framing and AI has been discussed to some extent; specifically, the issue of interpretation has been raised as a major concern; AI will interpret the goal differently than the human programmer intended to. But the concept of framing is also relevant for public policy on AI. For example, governance in AI policy documents can be framed (in the ‘wide’ sense) to resolve public controversies regarding AI (Ulnicane et al. 2020). Perry and Uuk note that the question of framing is essential for AI governance as well as for whether issues are considered to be policy problems or not (Perry and Uuk 2019). They argue that the AI governance community needs to think about how issues are framed, and subsequently, the consequences of a particular framing.

Concerns over letting the tech industry set and drive the agenda for AI policy and the extent of its influence over ethical AI regulation have already been raised (Cath 2018; Reinecke et al. 2021). The private sector is involved in developing regulation for AI both by direct participation, such as in the EU High-Level Expert Group on AI where almost half the representatives were from the industry, or by lobbying efforts. Whether intentional or not, the tech industry promotes and encourages certain conceptions and perceptions of what AI is; AI technology and development as unavoidable and necessary for economic development and growth since it will lead to efficiently gains while potential harms can be mitigated. The global initiatives on AI ethics are also influenced by the private sector (Nemitz 2018). According to Nemitz, such influence can result in too narrow understandings of accountability, fairness and transparency.

Considering the gap between what is currently possible and what can be imagined, much of the discourse relies on the potential of AI, making framing highly significant. For instance, Elish and Boyd note that the capabilities of systems such as Watson or AlphaGo are quite narrow, contrary to what some of the hype might suggest (Elish and Boyd 2017). They argue that “the narratives around such games, when they are performed for a public audience, serve to obfuscate the true state of the field” (ibid). While a certain degree of hype can be necessary for innovation, Elish and Boyd point out that the frame promoted by the industry “encourages a specific interpretation of what Watson is” (ibid). Obviously, AI governance is shaped by how AI is understood and imagined. The issue can even be seen in the term ‘AI solutions’ used by the HLEG in their recommendations. Whereas the term ‘system’ is more neutral, the term ‘solution’ indicates that there is a problem and that it will be solved.

Veale discusses framing issues and points out that the term AI has become ambiguous and general, with AI often being indistinguishable from computing or statistics (Veale 2020). Further, Veale notes that policies often do not sufficiently recognize the importance of problem structuring and framing. Sometimes, other solutions to societal problems can be ‘better’ compared to rushing to implement AI. Instead of identifying possible areas where AI can make government more efficient, we might as well identify areas where there is no need for ‘AI solutions’, perhaps because there is no problem to begin with.

Similarly, another perspective related to regulation is that AI is in its essence just mathematics and statistics. Regulating algorithms can be framed as regulating equations or saying “you cannot use multiplication”. However, as Veale states “tools cannot define the problem they are applied to” (Veale 2020). If AI is seen as a tool, the implication is to focus regulation on user cases and factual applications of AI. For example, “AI should not be weaponized” or “face-recognition should not be used as part of general surveillance”.^12^ Framing AI as general technology would yield considerably different from policies considering the specific applications, or use-cases, of AI. Perhaps both views are needed but the frame of AI as computation and mathematics can hinder regulation of the technology, such as requiring AI in general to be explainable, fair and accountable.

Lastly, it is essential to note that the concept of framing in a justificatory role can be used to analyze how policy decisions on AI are being justified. The optimistic framing of AI technology and the promise of efficiency and fairness (European Commission Independent High-Level Expert Group on Artificial Intelligence 2019) can, as argued by Elish and Boyd “obscure the limitations of the field and trade-offs involved in doing technical work under the rubric of AI”. The current hype can contribute to the hasty implementation of AI systems in the public domain, without recognizing the specific challenges of AI for public use.

### Temporal strategies

Public policy decisions regarding AI could be made more approachable by adopting clear temporal strategies. Given the complexities and uncertainties involved, decision-makers could benefit from (for example) using the framework for systematic deliberation proposed by Gertrude Hirsch Hadorn (2016). By intentionally extending decision-making over time, decision-makers can learn about, evaluate and account for the uncertainties at hand. Moreover, temporal strategies facilitate the adaptation of revisions of the framing of certain components of the decision problem as well as reconsideration of the arguments for and against options for choice.

There are three temporal strategies that could be considered in the context of decision-making on AI policy: (i) decisions could be postponed, (ii) made recurrently, or (iii) sequentially (ibid). Postponement, the first strategy, can be made both passively (“wait and see”) and actively—when measures are taken to search for additional information. The purpose of a postponement of a decision about public policy is to get more information and is sometimes called a “moratorium”. For example, a resolution by the European Parliament invites the European Commission to consider a moratorium on the use of facial recognition systems in public spaces by public authorities and spaces for education and healthcare

“*until the technical standards can be considered fully fundamental rights-compliant, the results derived are non-biased and non-discriminatory, and there are strict safeguards against misuse that ensure the necessity and proportionality of using such technologies*” (European Parliament Resolution 2021).

However, it should be noted that postponement does not guarantee that a decision under uncertainty in time (and with additional information) will become a decision under certainty. Thus, a decision-maker has to evaluate if time will reasonably affect the decision that has to be made. Given the inherent uncertainty of AI, adopting either passive or active postponement as the only policy is not recommended since significant uncertainty will remain even if we allow some time to pass, and some of the uncertainty might diminish.

A second possible strategy is so-called semi-closure, which allows a decision to be taken recurrently. Here, a provisionary decision is taken and reconsidered when some time has passed. The strategy can also be applied to take decisions on parts of a problem successively. While this adaptive strategy can seem persuasive, it needs to be recognized that leaving a decision open for reconsideration can give rise to uncertainties and a lack of commitment to implement the policy (Edvardsson Björnberg 2016). This needs to be kept in mind when considering adaptive governance (similarly to what is being proposed by Wallach and Marchant (2019) but more explicit) in the context of AI policy. This particular strategy can be used to downscale decisions, use participatory approaches and interpretive methods to understand and adapt policy. Such as strategy is an approach of many ‘decisions and revisions’ and could, thus, answer the need for quick adaptation as suggested necessary for AI policy. Considering it is also a strategy that is suitable for decisions with inherent variability (Hirsch Hadorn 2016), applying it in the context of AI seems to have potential.

The last possible strategy is making sequential decisions, sometimes called ‘dynamic choice’ (ibid). This strategy can include both postponement and semi-closure and is both more complex and more flexible than the two previously discussed strategies. A strategy of sequential decisions can also provide guidance on how to formulate decisions on AI policy, such as identifying the proper framing and specifications of decisions, identifying what information is needed and considering future decisions linked to the current one. Unsurprisingly, past decisions need to be considered in planning ahead. Moreover, decisions could be partitioned into parts. Given the complexity of AI, striving for a comprehensive policy on AI is perhaps too ambitious, while policy on implementations of AI systems for public purposes is more feasible. Such a policy could (and should?) be open to learning, evaluation and account for uncertainty. To maintain stability, criteria for revisions and reconsiderations should be established. This is part of the governance that is needed when adopted a temporal strategy.

## Conclusions

Based on decisions regarding AI policy being decisions under great uncertainty, I have proposed applying elements of the argumentative approach to mitigate the challenges this poses for decision-makers. The perspective of framing is especially relevant since it could be argued that a particular frame already has been established. As argued by Cath (2018) and echoed by Reinecke et al. (2021), the private sector and its experts have been allowed to set the tone, framing AI technology as not only positive (despite great uncertainties) but also necessary for economic growth and prosperity. As such, public policies are set out to allow as much as possible while regulating only to minimize risk. This regulatory perspective can be contrasted to other technologies with great uncertainties such as GMO and CRISPR where the regulatory approach has been much more cautious, regardless of potential societal benefits.^13^

Given the lack of information on alternatives and outcomes, alternative strategies for decision-making on AI policies for public use should be utilized. Instead of aiming for overarching AI policies, challenging questions could be divided into smaller parts. For example, policies on the implementation of AI in the public sector could be adopted prior to regulating policies on AI in general by a ‘divide and conquer’ approach. Taking into account the potential harm wrongful implementations of AI for public use can have, a cautious approach should be taken. While it could be claimed this would hinder innovation, it should be acknowledged that more is required of AI in the public domain than efficiency and innovation. Given the conclusions of this paper, the uncertainty in the realm of AI policy is not going away. Further work can lead to better understanding of the implications of uncertainty for particular AI applications. In the meantime, AI policies need to acknowledge and take into account the uncertainty at hand.

## Footnotes

1 The argumentative approach is decision theory is unrelated to *“*The argumentative turn in policy analysis and planning” edited by Fisher and Forester (1993), even though it advocates similar approaches in arguably adjacent disciplines.

2 By ‘AI for public use’ I refer to applications of AI in the public sector. The public sector consists of governments, publicly controlled agencies and other entities that deliver public programs, goods or services such as the police, military, public education and public transport among many others.

3 A discussion on whether this implies that machines are rational agents, have intentions and/or desires is out of scope for this paper.

4 A narrow implementation of AI has a narrow goal, such as ‘following the rules of chess, make a sequence of moves to secure a win’. In comparison, a general AI does not have such narrow goals and is thus closer to our intuitive understanding of ‘intelligence’.

5 I take the term uncertainty to be value-neutral (in contrast to the term risk, which usually refers to something unwanted). However, there are normative aspects to the strategies discussed in Sect. 7. For example, a framing analysis can make hierarchies of values explicit. Additionally, decisions on policies are inherently normative since they dictate to whether a course of action is permissible or mandatory.

6 The question of whether or not XAI is achievable or even necessary is out of scope for this paper; see Arrieta et al. (2020) for more on explainable AI.

7 Also, see work by Tim Dutton (2018) for an overview of National AI Strategies.

8 This is not to be confused with work on guidelines for AI as such.

9 The difficulty in avoiding certain criteria to be identified as relevant was why Amazon discontinued their HR implementation of AI. There was simply no way to make the algorithm not favor men in the application process (https://www.reuters.com/article/us-amazon-com-jobs-automation-insight/amazon-scraps-secret-ai-recruiting-tool-that-showed-bias-against-women-idUSKCN1MK08G).

10 While there are many different uses of the concept ‘rationality’, it is well-specified and well-understood in the discipline of decision theory (Jones 1994). According to Jones, the rational choice approach thrives in all major social science disciplines due to features of coherency and deductively, despite being criticized. Among critics, a commonly proposed alternative is bounded rationality (Simon 1979).

11 In the context of AI and data protection, this could be exemplified by the difference between the default of opt-out versus opt-out whenever you are prompted to share certain data online. Suppose that people generally prefer to keep their data private. In that case, the question of whether you wish to share data could be formulated differently. Namely, it is possible present the user with an opt-in alternative (and assume opt-out prior to choice) rather than presume opt-in if no choice is made.

12 Such regulation has been adopted in San Francisco: https://www.nytimes.com/2019/05/14/us/facial-recognition-ban-san-francisco.html

13 More speculation on the difference between AI and (for example) GMO is that the opponents of GMO having been able to present the risk as both relevant and serious while those opposed AI are presenting risks that seem like science-fiction (think “general AI taking over the world”).
